# Induction of potentially lethal hypermagnesemia, ischemic colitis, and toxic megacolon by a preoperative mechanical bowel preparation: report of a case

**DOI:** 10.1186/s40792-016-0145-6

**Published:** 2016-02-20

**Authors:** Masahiko Sugiyama, Eiji Kusumoto, Mitsuhiko Ota, Yasue Kimura, Norifumi Tsutsumi, Eiji Oki, Yoshihisa Sakaguchi, Tetsuya Kusumoto, Koji Ikejiri, Yoshihiko Maehara

**Affiliations:** Department of Gastroenterological Surgery, Clinical Research Institute Cancer Research Division, National Kyushu Medical Center, Fukuoka, Japan; Department of Surgery and Science, Graduate School of Medical Sciences, Kyushu University, 3-1-1 Maidashi, Higashi-ku, Fukuoka, 812-8582 Japan

**Keywords:** Mechanical bowel preparation, Hypermagnesemia, Ischemic colitis, Toxic megacolon

## Abstract

A 67-year-old man was diagnosed with rectal cancer. The tumor invaded the subserosal layer, but it was not large, and there was no sign of obstruction. Neo-adjuvant chemotherapy reduced the size of the tumor. The patient was admitted to our hospital for surgery. For mechanical bowel preparation, he ingested 34 g of magnesium citrate (Magcorol P®), but then developed severe shock, a disturbance of consciousness, and acidemia, and he required catecholamines and mechanical ventilation. X-ray, CT, and laboratory tests revealed ischemic colitis, toxic megacolon, and hypermagnesemia (16.3 mg/dL). After 2 days of temporary hemodialysis and an enema to reduce his blood magnesium concentration, he recovered and left the intensive care unit. However, the left side of his colon had suffered ischemic damage and become irreversibly atrophied. One month later, he underwent laparoscopic abdominoperineal resection and left-side colectomy for the rectal cancer and severe ischemic colitis of the left side of the colon. Histopathology confirmed the rectal cancer with a grade 2 chemotherapeutic effect and severe ischemic colitis of the left side of the colon. Hence, the present case suggests that severe ischemic colitis, toxic megacolon, and hypermagnesemia can occur after taking a magnesium laxative without obstruction of the intestine.

## Background

Hypermagnesemia is considered to be very rare [1]; it is usually caused by a laxative overdose, and it may occur in patients with kidney dysfunction. In many cases, severe shock occurs; thus, early diagnosis and proper therapy are necessary to save the patient’s life. Ischemic colitis is caused by a reduction in blood flow to the colon [2]. Patient diagnosis and treatment can be challenging, since colonic ischemia often occurs in patients who are debilitated and have multiple medical problems. Toxic megacolon is a potentially lethal complication of inflammatory bowel disease (IBD) or colitis due to *Clostridium difficile* infection, which is characterized by total or segmental nonobstructive colonic dilatation plus systemic toxicity [3–5].

We present a case of a patient with potentially lethal hypermagnesemia accompanied by ischemic colitis and toxic megacolon who took a laxative as part of surgical pretreatment. To the best of our knowledge, this is the first report in the literature of a triplet of hypermagnesemia, ischemic colitis, and toxic megacolon resulting from ingestion of a preoperative laxative.

## Case presentation

**Fig. 1:**
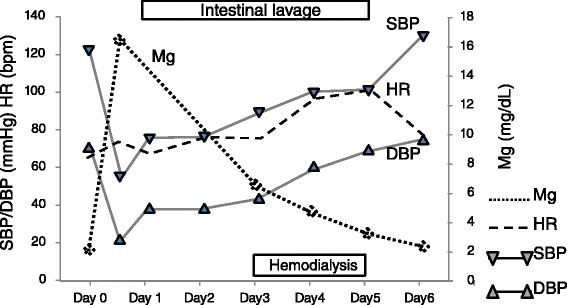
Clinical course and value of the patient. *Mg* magnesium, *HR* heart rate, *SBP* systolic blood pressure, *DBP* diastolic blood pressure

**Fig. 2:**
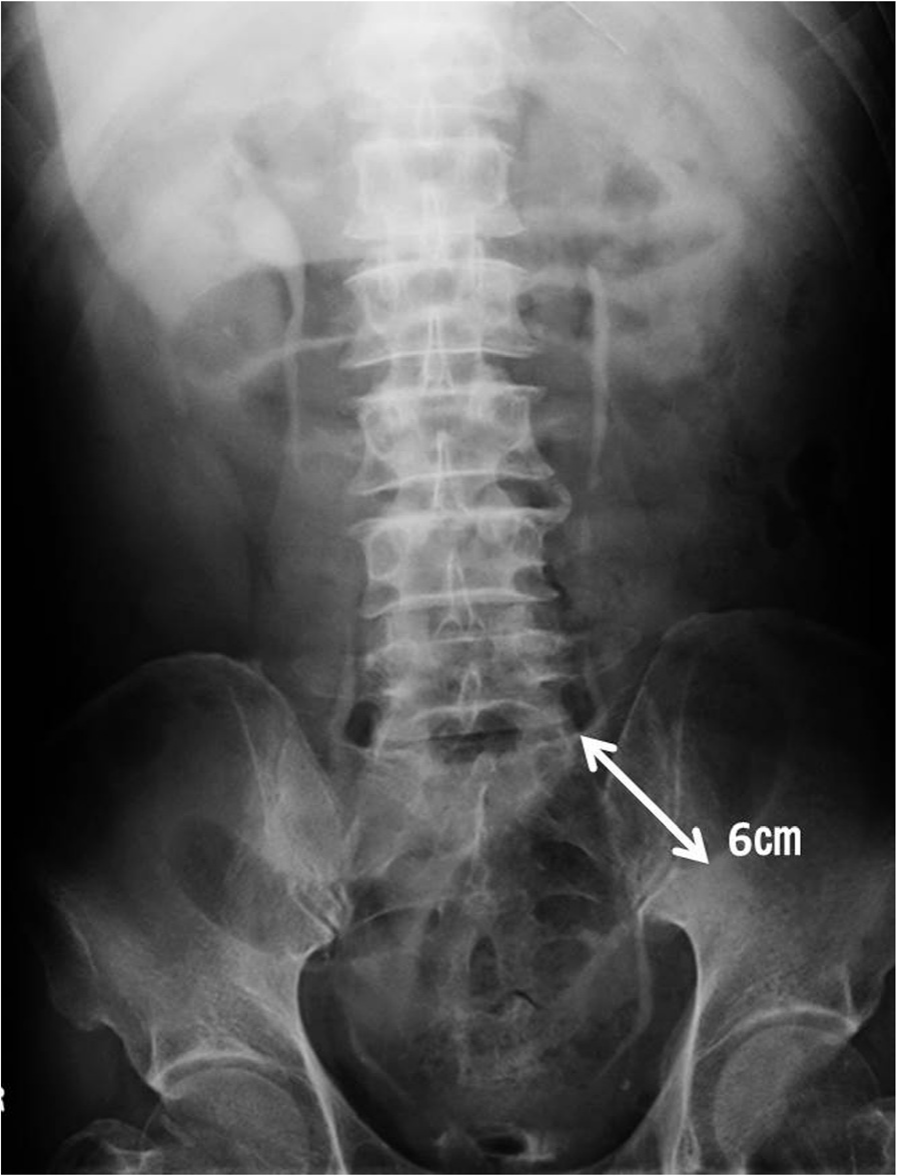
Abdominal X-ray examination revealed 6-cm dilatation of the sigmoid colon with fecal impaction

**Fig. 3:**
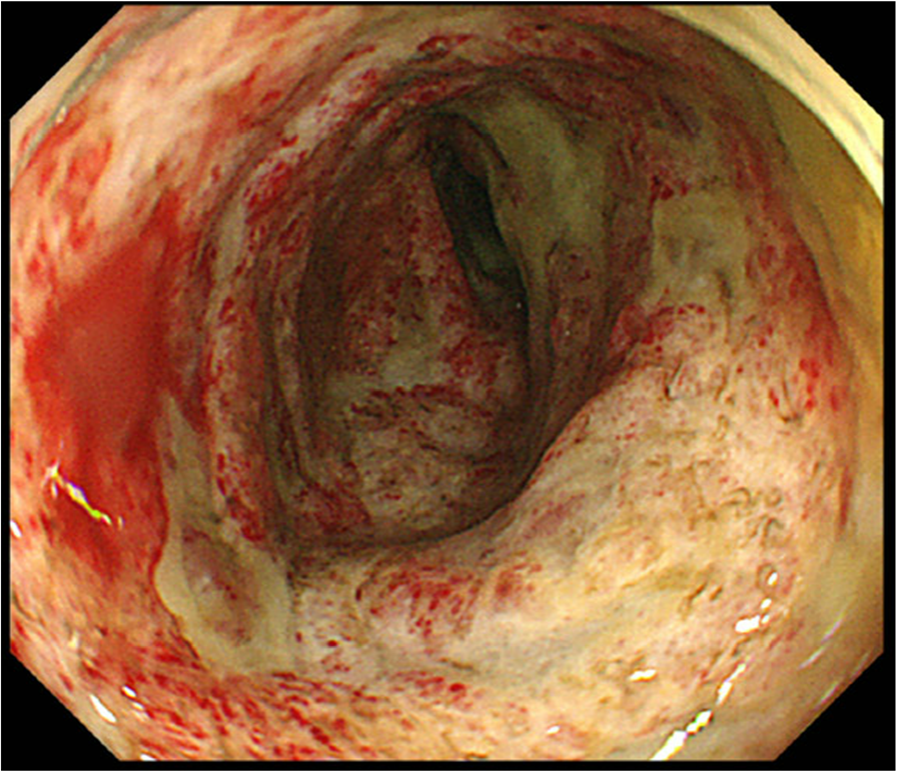
Colonoscopy showed a severe ulcer and pseudomembrane at the descending colon

**Fig. 4:**
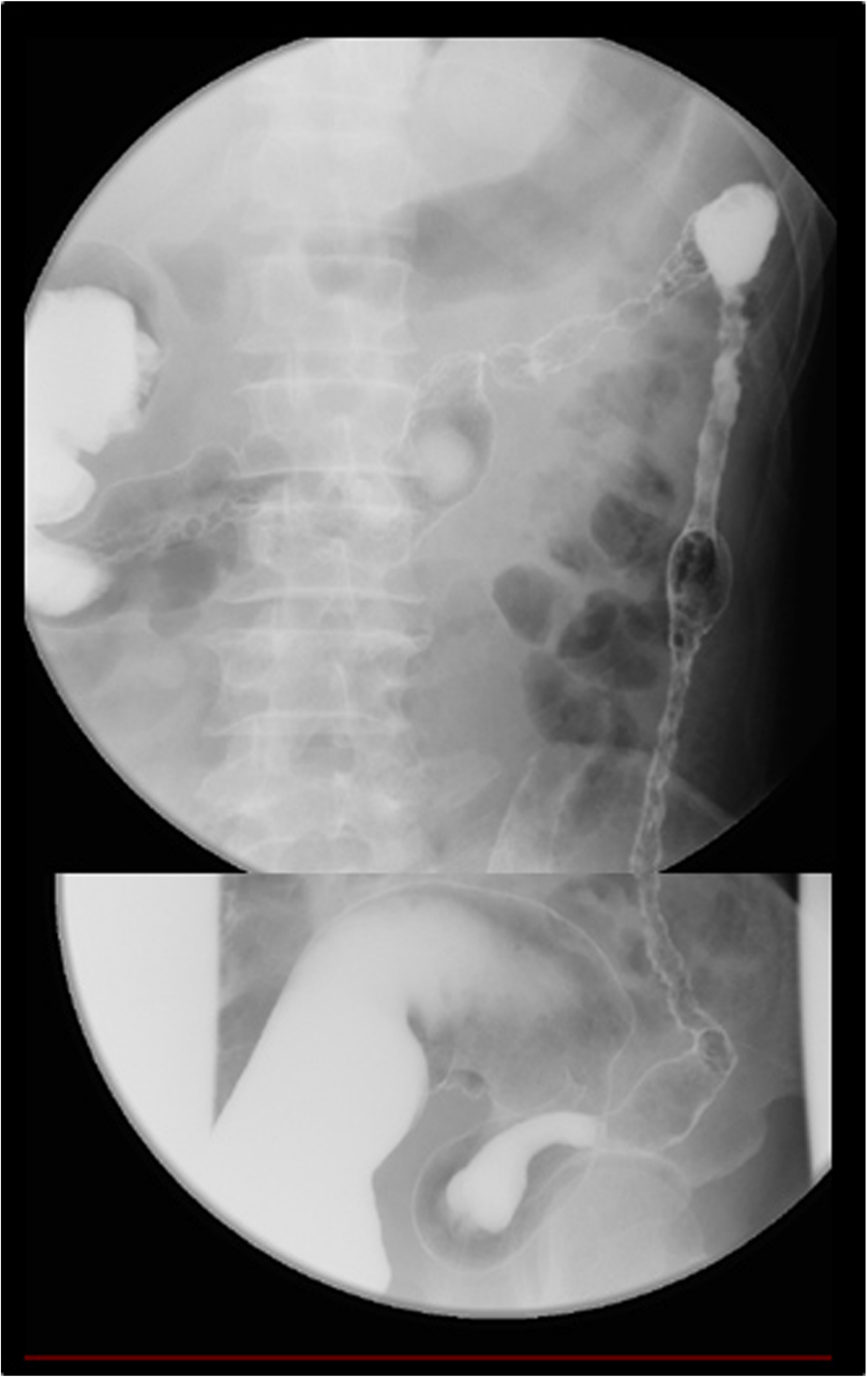
Contrast enema examination revealed significant stenosis on the left side of the transverse colon to the descending colon

**Fig. 5:**
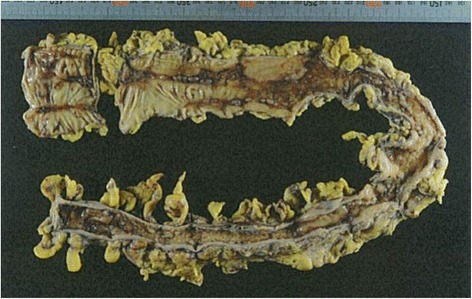
Pathological findings showed advanced rectal cancer and significant atrophic changes and a longitudinal ulcer at the transverse colon and descending colon. The findings were compatible to ischemic colitis

The patient was a 67-year-old man. He had experienced bloody stool and anal pain for 1 year and then visited our hospital. Colonoscopic examination showed a locally advanced type 2 rectal tumor that had invaded the subserosal layer at the Rb portion, and a CT scan revealed several swollen lymph nodes. There was no sign of metastasis to other organs. The patient’s past medical history was significant for hypertension and slight renal dysfunction (blood urea nitrogen (BUN), 10 mg/dL; creatinine, 1.23 mg/dL; and eGFR was 52.8, chronic kidney disease (CKD) stage 3a). To reduce the size of the tumor, we treated the patient with four courses of neo-adjuvant chemotherapy with the SOX regimen (TS-1 120 mg/body and oxaliplatin 80 mg/m^2^), and he completed the treatment without any adverse events. Preoperative examination revealed that the tumor had decreased more than 30 % in diameter, and the response was regarded as a “partial response” by RECIST criteria [6]. There was no obstruction, and minimal tumor at the rectum by colonoscopy, and his nodes had also decreased by CT scan. He was admitted to undergo a laparoscopic abdominoperineal resection, and, for mechanical bowel preparation (MBP), he received 34 g of magnesium citrate (Magcorol P®; containing 2.71 g of magnesium (Mg)) orally at 2:00 PM, day 0. Three hours later, he had only scant excrement, and he felt abdominal distention and muscle weakness in his legs. In spite of additional anthraquinone laxatives, he could not get excrement. On day 1, at 2:00 AM, he was lethargic and did not respond well to verbal stimuli. His systolic blood pressure (BP) was less than 55 mmHg, and his heart rate (HR) was 75/min (Fig. 2). The electrocardiogram showed the findings of first-degree atrioventricular block, prolonged PR interval (0.281 ms), and slightly prolonged QRS interval (0.118 ms). Intravenous epinephrine and dopamine were started to restore normal BP, but the effect was limited, and the BP soon fell again. Next, the patient was transferred to the intensive care unit (ICU) and was intubated. Under dopamine (10 μg/kg/min), dobutamine (5 μg/kg/min), and norepinephrine (0.7 μg/kg/min), his systolic BP was 75/40 mmHg, his HR was 70/min, and his abdomen was soft but distended and tympanic, but there was no muscular defense. Emergency CT and abdominal X-ray examinations revealed diffuse and marked dilatation of the large-bowel loops, with fecal impaction 6 cm in diameter at the sigmoid colon (Fig. 1), and no free air in the abdominal cavity. His head CT scan was of normal finding, as was his echocardiography findings. Digital rectal palpation revealed no anal or rectal stenosis, and there was enough room to pass the stool. We drained the fecal impaction by intestinal lavage and a transanal tube (12 Fr silicone tube). A stool culture revealed normal flora and no *C. difficile* or other toxigenic bacteria. A large volume of liquid excrement was removed, but his abdomen was still distended, and his BP was still low, even though several catecholamines were on board. At first, our diagnosis was functional fecal ileus with an infection due to “bacterial translocation,” and we thought that the patient developed septic shock. Despite his markedly low BP, his HR was not increased; relative bradycardia continued. Then, the presence of hypermagnesemia was suspected. The patient’s serum Mg level on day 3 was 6.4 mg/dL, and blood drawn at day 1 showed a markedly elevated serum Mg concentration of 16.3 mg/dL (Fig. 2). The serum Mg level at admission was normal (1.8 mg/dL), and the diagnosis of symptomatic hypermagnesemia was confirmed. Calcium gluconate was infused to antagonize the effects of hypermagnesemia. To reduce the serum Mg concentration, we performed temporary hemodialysis. After 2 days, hemodialysis and daily intestinal lavage enabled the serum Mg level to decrease to almost the standard level, and the patient’s hemodynamics also dramatically improved. Intravenous catecholamines were gradually withdrawn, and the patient was extubated at day 5. Though his hemodynamics were improved, and the inflammation was gone, his abdomen remained distended. On day 15, colonoscopy revealed that the mucous membrane was sloughed and the sigmoid colon had an ulcer that covered the entire circumference, similar to the severe stage of IBD (Fig. 3). The examination could not progress beyond the sigmoid descending colon junction because stenosis of the descending colon made it too tight for the scope to pass. On day 32, a contrast enema examination revealed notable stenosis on the left side of the transverse colon to the descending colon (Fig. 4). These findings confirmed the diagnosis of ischemic colitis. The patient tried oral intake, but just a liquid nutritional supplement caused a stomachache and bloody diarrhea. Subsequently, he ingested only liquid water and was given intravenous alimentation. While waiting for recovery of his general condition, on day 52, he underwent laparoscopic abdominoperineal resection with left-side colectomy with D3 lymph node dissection. Laparoscopic observation revealed that the entire transverse colon and descending colon were severely narrowed. Pathological findings showed advanced rectal cancer, and at the transverse colon and descending colon, a longitudinal ulcer on the opposite of the mesenteric attached side, and severe stenosis with massive invasion of inflammatory cells within the whole thickness of the colon wall. The findings were comparable to those of ischemic colitis (Fig. 5). The patient had a good postoperative course and was able to eat normal foods. He was discharged on day 72 (postoperative day 20) without any postoperative complications.

### Discussion

Magnesium (Mg) is the fourth most abundant cation in the human body and the second most abundant intracellular cation, with 67 % in bone, 31 % in intracellular spaces, and 1–2 % found extracellularly [7]. Mg homeostasis is dependent mainly on gastrointestinal absorption and renal excretion, and the kidney is the main organ involved in Mg regulation. Renal Mg excretion is very efficient because the thick ascending limb of Henle has the capacity to completely reject Mg reabsorption under conditions of hypermagnesemia [8]. Even in the presence of decreased renal function, the serum Mg level is regulated by a reduction in the gastrointestinal absorption of Mg [9]. Therefore, hypermagnesemia is most frequently seen in conjunction with renal insufficiency and ingestion of Mg-containing drugs or in patients with small-bowel hypomotility disorder. Moreover, hypermagnesemia can decrease bowel motility by blocking myenteric neurons and interfering with excitation-contraction coupling of smooth muscle cells [10]. In addition, hypermagnesemia can block the peripheral and autonomic nervous systems via anatomization of calcium effects, suppression of acetylcholine release, and reduction of postsynaptic membrane responsiveness and depress the conduction system of the heart and sympathetic ganglia [11]. Thus, the clinical manifestations of hypermagnesemia vary according to the serum Mg concentration. Hypotension, nausea, vomiting, facial flushing, urinary retention, and ileus occur at levels ranging from 5 to 8 mg/dL, while the absence of the deep tendon reflex and somnolence and complete heart block occur at 9–12 mg/dL. Respiratory depression, paralysis, and complete heart block occur at levels >15 mg/dL, and cardiac arrest occurs in asystole at levels >20 mg/dL [12, 13]. However, the peak serum Mg concentration does not correlate with mortality or the severity of intestinal complications [14, 15]. Although our case showed some manifestations of severe hypermagnesemia, we did not recognize these manifestations until day 3 because his clinical manifestations were concurrence of hypermagnesemia, ischemic colitis, and severe shock. Equivalent to CKD stage 3a, during preoperative chemotherapy in our case, renal function was maintained, and there were no abnormalities of serum electrolytes, even Mg. The patient presented with a serum creatinine level of 1.19 mg/dL on day 0 before laxative intake, which was a completely normal value at hospitalization, and there was no stenosis, including rectal, at the site of the tumor. However, a Mg laxative administered as part of the preoperative preparations reduced and weakened his colonic motility in inverse proportions with increased serum Mg, and his colon became dilated such that a lot of Mg might have been absorbed. Finally, the level to which the serum Mg had increased became critical. Treatment for hypermagnesemia requires increasing renal Mg excretion through intravenous diuretics, and sometimes, hemodialysis is effective when kidney function is impaired and the patient is symptomatic from hypermagnesemia. In this case, drainage of fecal impaction by intestinal lavage was also effective to reduce the intra-bowel Mg concentration.

Distention of the colon due to decreased bowel motility could lead to continuous ischemia of the entire thickness of the bowel wall and ultimately induce necrosis of a part of the colon. Ischemia due to dilation of the colon may be provoked when there is an increase in intraluminal pressure to more than 50 mmHg [16]. Acute colonic ischemia has a high mortality rate, depending on the cause of the event, the degree of ischemia, and the extent of ischemic bowel. Ischemic colitis is likely to develop in the left colon, including the splenic flexure and sigmoid colon, because of its arterial anatomical features. In our case, the left side of the transverse colon and the descending colon became gangrenous, as ischemic colitis, despite the fact that the sigmoid colon, which is located anatomically close to the inferior mesenteric artery, showed normal findings.

Toxic megacolon is a rare but severe and potentially fatal complication of colonic inflammation. Most often, it is associated with IBD [17] specifically ulcerative colitis and, to a much lesser extent, Crohn’s disease [18]. Ischemia and amebic colitis may also produce a toxic megacolon pattern [19]. Its main characteristics are radiographic evidence of total or segmental colonic distension of >6 cm. In this case, the patient had received an amount of magnesium sodium, and despite the fact that it was the result of laxative intake, he could not use his bowels. Orally ingested Mg had stayed in the intestine much longer, and intestinal absorption of Mg might have been enhanced. Initially, hypermagnesemia occurred and caused bowel hypomotility. Prolonged hypotension and a pressure effect of fecal impaction on the intestinal circulation could have induced intestinal ischemia, and the patient developed toxic megacolon. Although a portion of the toxic megacolon recovered from a severe condition to almost normal digestive function, the damage to his left colon was serious, and hard sclerotic changes occurred. Finally, he required resection, and surgery for rectal cancer was performed simultaneously. To our knowledge, this is the first case report to show that administration of mechanical bowel preparation can lead to the development of three rare complications: hypermagnesemia, ischemic colitis, and toxic megacolon.

## Conclusions

This case shows that preoperative mechanical bowel preparation containing magnesium sodium might result in hypermagnesemia regardless of stenosis and it may cause life-threatening events, such as ischemic colitis and toxic megacolon.

## Consent

Written informed consent was obtained from the patient for publication of this case report and any accompanying images. A copy of the written consent is available for review by the Editor-in-Chief of this journal.

## Abbreviations

IBD: Inflammatory bowel disease
